# Electronic screening for lifestyle issues and mental health in youth: a community-based participatory research approach

**DOI:** 10.1186/s12911-016-0379-z

**Published:** 2016-11-08

**Authors:** Felicity Goodyear-Smith, Arden Corter, Hannah Suh

**Affiliations:** Department of General Practice & Primary Health Care, University of Auckland, PB 92109, Auckland, 1142 New Zealand

**Keywords:** Medical informatics, Clinical decision making, Community-based participatory research, Adolescent, Mental health, Risk reduction behavior, Help-seeking behavior, Implementation, Patient participation, Psychosocial deprivation

## Abstract

**Background:**

We previously developed YouthCHAT, a youth programme for electronic screening and intervention for lifestyle risk factors and mental health issues. Our aim was to tailor the YouthCHAT package for use in a clinic catering for disadvantaged youth, assess its acceptability and utility, and develop a framework to scale-up its implementation.

**Methods:**

We used a community-based participatory research approach to implement YouthCHAT in a rural clinic in New Zealand. Modifications to the programme were developed using an iterative process involving clinicians and patients. Electronic YouthCHAT data were collated and descriptive statistics produced. Quantitative data from post-consultation youth surveys were analysed, with thematic analyses undertaken of free text responses and staff interviews. A generic implementation framework was developed with modifiable components.

**Results:**

Thirty youth, predominantly female Māori, completed electronic screening then attended their clinician. Consultations included discussion of YouthCHAT responses, with joint problem-solving and decision-making regarding intervention. Twenty-seven (90 %) screened positive for at least one domain. Nineteen (67 %) had one to three issues. Sixteen (53 %) wanted help with at least one issue, either immediately or later. Patients gave YouthCHAT high acceptability ratings (*M* = 8.29/10), indicating it was easy to use, helped them think about and identify problems, talk with their doctor, and assisted their doctor to be aware of these issues. They liked that YouthCHAT kept them busy in the waiting room and gave them time to reflect on their responses, and what to discuss with their clinician. Clinicians felt that YouthCHAT was acceptable to their young patients because it was electronic and reinforced their privacy. They indicated YouthCHAT identified problems that would have not been identified in a normal consult, and improved consultations by making them faster. The clinic continues to use YouthCHAT post-study.

**Conclusions:**

A community-based participatory approach was used to engage key stakeholders (patients and clinic staff) for ‘real life’ translation of an electronic mental health and lifestyle screening and intervention package into a specific youth clinic context. Patients and staff found the programme acceptable and useful, and a framework was developed for scaled up and sustainable tailored implementation in other settings.

## Background

Youth mental health and risky behaviour problems including substance misuse, depression, anxiety, anger and abuse are common in New Zealand, leading to significant personal, social and economic consequences. Research shows that 27 % of students are affected by anxiety and depression, with the greatest growth in prevalence between ages 15–18 [1]. Hazardous drinking exceeds 50 % prevalence at age 18. New Zealand has a high rate of suicide for males aged 15–19, with Māori males living in deprived areas having the highest rates.

Inequalities in health and social outcomes such as suicide and domestic violence mean that Māori are less likely than Pakeha [2], and youth less likely than adult [3], to access needed healthcare. Reasons include factors such as shame, lack of service awareness, services not appropriately targeted, poor insight, and in some cases conditions like depression make it difficult to seek support.

Current school-based support services are unsystematic, meaning many students miss getting needed support and interventions [4]. The World Health Organization recognises the need for appropriately targeted services to address the unique health and social needs of youth – services that are easy for youth to access, and which provide appropriate tools [5]. Youth want a greater say in how services are designed and delivered, and expect services to be diverse, contemporary and responsive.

A comprehensive and validated screening programme is needed in school-based and primary health settings, enabling detection of and early intervention for vulnerable youth [6, 7]. The validated New Zealand instrument eCHAT (electronic Case-finding and Help Assessment Tool) [8, 9] assesses behaviours (smoking, drinking, other drug use, gambling, physical inactivity, being subjected to abuse) and negative mood states (anxiety, depression, anger) that impact on health, allows patients to indicate whether they would like help, and prioritises the type of help needed [10, 11]. It is self-administered electronically with a summary provided to the family physician or nurse, in order for identified issues to be discussed during consultation with shared decision-making on what interventions and courses of action to pursue.

A youth version (YouthCHAT) has been developed including a Māori language (Te Reo) version, sexual health questions (on concerns about orientation, risky behaviour or unwanted sex); and the Alcohol, Smoking and Substance Involvement Screening Test (ASSIST) for alcohol and drugs [12] replaced with the youth-friendly, validated Substances and Choices Scale (SACS) [13]. Four additional screening tools are activated when a positive response is triggered by the patient: ASSIST for smoking; SACS; Patient Health Questionnaire for Depression (PHQ-9) [14], and Generalised Anxiety Disorder screen (GAD-7) [15]. A ‘Help’ question is also triggered at the end of each positively responded domain for patients to alert clinicians of their readiness for change.

## Methods

The aims of this study were to pilot the YouthCHAT program, assess its utility and acceptability for both enrolled and non-school enrolled youth and for health clinic staff, and build a framework for subsequent roll-out.

YouthCHAT was implemented in a health clinic co-located at a low-decile school with a high Māori population in rural New Zealand. Fifty percent who attend the clinic are non-school enrolled Māori youth, including teenage parents and unemployed. We used an organic iterative approach to implementing the programme, identifying processes in consultation with clinical staff and patients. Adjustments were made to the programme made in response to feedback at different times, depending on the nature of the issue. Changes to delivery processes were in response to local need.

Community members proficient in Te reo provided Māori translation of all YouthCHAT questions, which were then back-translated for validation and programmed in. A YouthCHAT user manual was developed in conjunction with practice staff with local community agencies and resources added. Information technology systems were field-tested, and data collection ran from November 2015 to January 2016. YouthCHAT questions were delivered to youth on an e-tablet. Screening results and scores were available immediately through secure transferral to the clinic’s electronic medical record (EMR) via a secure server. Clinic staff reviewed YouthCHAT results to identify youth in need of immediate help (e.g. triggered a self-harm alert) and/or who had scored positively for issues measured by YouthCHAT (e.g. substance abuse) and who wanted help.

Resources to guide interventions for each domain were compiled in conjunction with clinic staff, using the stepped care approach of: self-management (helplines, handouts, websites and e-therapy); clinician-provided brief interventions and medications and local community agencies and support services; and mental health and drug and alcohol secondary care services.

All recruited youth were invited to complete a survey after their consultation. We used a mixed methods study design. Measures included demographics (age, gender, ethnicity, employment status), number of YouthCHAT issues for which youth wanted help, Likert scores for acceptability and utility, and free text comments.

The questions were based on those used for eCHAT studies in different contexts, plus generic questions used in other studies reported in the literature. The format and wording of the questioning was modified in response to informal feedback on youth-friendly language from adolescents. For example, in a 10-point likert scale on how they found YouthCHAT, the options ranged from lame to awesome. The questionnaire hence had face validity, although not formal criterion-based validity.

A focus group of patients was held to elicit feedback and ideas for improving the system. Feedback from staff was obtained through semi-structured interviews. Audiotapes were confidentially transcribed.

Descriptive statistics were analysed using Excel and SPSS. Qualitative data underwent thematic analysis using a general inductive approach. Ethical approach was obtained through the Health and Disability Ethics Committee (NTY/11/10/102/AM03). An amendment to cover this current study was obtained 09/2015.

We conducted an evaluation of our processes and developed a framework for further implementation and scaling up for delivery of the YouthCHAT programme.

## Results

All consecutive patients were recruited to participate during clinic time. There were no declines, but one response was excluded because an age >65 years was wrongly entered on YouthCHAT. Thirty patient participants under age 25 years completed YouthCHAT and the survey. Twenty-eight (93 %) were female, and 27 (90 %) were Māori, with the remainder NZ European. Twenty (67 %) were students, four (13 %) were employed, five (16 %) were unemployed, and one was a parent. Five participated in the focus group discussion (P1–P5). Practice staff (family physician and nurse) underwent semi-structured interviews (S1-S2).

### Responses to YouthCHAT

The number of positive responses and those wanting help, either during the ensuing consultation or at a later time, are recorded in Table 1.

**Table 1:** Number of positive responses and help-seeking for each domain

YouthCHAT domain	Positive *n* (%)	Wants help^a^ *n* (%)	Wants help today^a^ *n* (%)	Wants help later^a^ *n* (%)
Smoking	13 (43)	3 (25)	1 (8)	2 (17)
Drinking or other drugs	23 (77)	5 (21)	1 (4)	4 (17)
Gambling	3 (10)	2 (66)	1 (33)	1 (33)
Depression	6 (20)	5 (84)	1 (17)	4 (67)
Anxiety	11 (37)	4 (36)	2 (18)	2 (18)
Sexual orientation	4 (13)	4 (100)	4 (100)	0
Sexually active	20 (67)	N/A	N/A	N/A
Risky sexual behaviour: STI	11 (55)	7 (64)	6 (55)	1 (9)
Risky sexual behaviour: pregnancy	7 (23)	5 (71)	5 (71)	0
Unwanted sex	5 (17)	2 (40)	0	2 (40)
Exposure to abuse	5 (17)	1 (17)	0	1 (17)
Anger control	13 (43)	8 (62)	2 (15)	6 (46)
Physical inactivity	13 (43)	4 (30)	2 (15)	2 (15)

^a^percentage of those who are positive

Positive responses regarding smoking, alcohol or other drug use, depression or anxiety triggers presentation of the added tools, ASSIST, SACS, PHQ-9 and GAD-7 respectively. Of the 12 (40 %) who smoked, nine had an ASSIST score indicating risk of health and other problems from current use, and three had scores indicating a high risk of experiencing severe problems (health, social, financial, legal, relationship) as a result of their current pattern of use, likely to be dependent. Of the 23 (77 %) who used alcohol or drugs, SACS scores indicated that seven needed further assessment, three had problems of clinical severity likely to need intervention, and six had serious problems likely to need secondary substance use services.

Six screened positive on the PHQ-9 for depression, two each for mild/moderate depression, and one each for moderately severe and severe depression. These latter two also triggered the PHQ-9 alert for possible self-harm. Three scored in the positive range for general anxiety disorder on the GAD-7.

YouthCHAT addresses 13 domains, although there is considerable overlap among domains of risky sexual behaviour, sexually transmitted infection and pregnancy. Only three youth were negative for all 13 domains, eight for one and eight for two, with the remaining 11 (37 %) ranging between three and nine positive conditions (Table 2). Fourteen indicated no issues with which they wanted help, five wanted help with one, four with two, and the remaining seven ranged between four and nine issues. However, for the majority of these, they only wanted help today for one problem, and indicated they would like help later for others, enabling a further consultation to be scheduled. The exception was one young person who wanted help during the ensuing consultation with smoking, drinking, risky sexual behaviour and anger control.

**Table 2:** Number of positive conditions indicated by respondents

Number of +ve conditions		Number	Percent
3	0	3	10
	1	8	27
	2	8	27
	3	3	10
	4	2	7
	5	2	7
	6	1	3
	7	1	3
	8	0	0
	9	2	7

Total number of conditions 13

### Acceptability of YouthCHAT

**Fig. 1:**
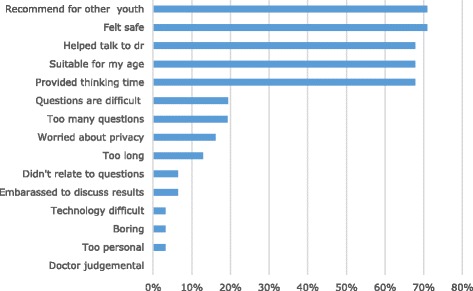
Acceptability ratings of YouthCHAT

Overall, youth gave high acceptability ratings (*M* = 8.29) where 1 = ‘lame’ and 10 = ‘awesome’. Most felt that YouthCHAT was appropriate for their age group and would recommend it to others (see Fig. 1). The majority said it helped them think about and identify problems and talk with their doctor, and it also helped their doctor be aware of these issues. Only a small minority had any objections to YouthCHAT questioning (e.g. questions too hard, too difficult or too many), and all in fact completed the entire questionnaire.

Analysis of the focus group and interview data identified similar themes (Table 2). Clinicians felt YouthCHAT was easy to use and the reports were user-friendly and straightforward. They thought that the screening tools were good and the summary scores helped guide their consultations. They also felt that YouthCHAT was acceptable to their young patients because it was electronic and reinforced their privacy. There were some concerns about the presentation of YouthCHAT – the interface was not appealing enough and the questions were too difficult for some youth.

### Utility of YouthCHAT

**Fig. 2:**
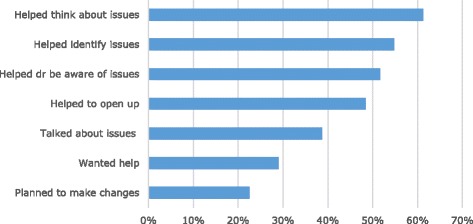
Utility ratings for YouthCHAT

YouthCHAT also scored highly with the patients on its utility (see Fig. 2). Qualitative results for utility are presented in Table 2. Most patients (*n* = 19) thought YouthCHAT helped them think about their health problems. Few wanted help with issues that YouthCHAT identified (*n* = 9). Although, 12 talked about their results with the doctor or nurse (*n* = 12), and seven made plans to address their problems.

Themes from qualitative analysis on YouthCHAT’s utility are presented in Table 3. Overall, as with survey data, results were positive. Patients liked the fact that YouthCHAT kept them busy in the waiting room, and gave them time to reflect on their responses and on what they might discuss with the clinician. Because of YouthCHAT, patients felt clinicians knew what they were concerned with before they walked through the clinic door, which facilitated discussion of the topics that YouthCHAT raised. Staff indicated that YouthCHAT identified problems that would have not been identified in a normal consult and reduced consultation time. Clinicians felt it was easier to open conversations and address certain issues via the results on the report, and hence improved problem-solving (Table 4).

**Table 3:** Themes identified in focus group and interviews with patients and staff regarding YouthCHAT acceptability

Theme	Example
Benefits	
Ease of Administration	“It was easy to use. We’re all used to the technology.” P3 “I thought it was going to be a little time consuming, but it was more smooth-running.” S1
Presentation (questionnaire)	“It was alright. Simple.” P2
Presentation (report)	“Layout was quite thorough and user-friendly” S1
Appropriate screening questions	“The questions weren’t difficult and were honest questions.” P2 “Screening tools were very good because they would formulate results onto the report and indicate to the clinician what level on the spectrum someone was on, which would then help the response of the clinician. That was beneficial” S2
Acceptability to youth	“Being that it is a tool used electronically, I knew being with young people, they are drawn towards using that or being open to using an electronic tool more so than a paper-based tool.” S2 It’s the same [opening up]. It’s just like talking to a screen instead a face. Makes no difference.” P3
Privacy/feeling comfortable	“When you’re answering questions on the iPad, it’s different to talking to a person, so you kind of feel more comfortable.” P1
Downsides/suggestions for improvement	
Presentation (questionnaire)	“Would like it with more colour.” P5 “I’d be interested to see whether there needs to be an inclusion of something more visual, more graphically pleasing so it engages them (youth) a bit more to the questionnaire.” S2 “A young person’s literacy ability and being unable to read … might need an audio option to help them answer the questionnaire.” S2
Nature of questions	“Some of the questions are hard and I didn’t understand them.” P5 “I think there were a little bit too many questions.” P2

**Table 4:** Themes identified in focus group and interviews with patients and staff regarding YouthCHAT utility

Theme	Example
Using waiting time	“Doing the YouthCHAT was way better in the waiting room than looking lost… Stopped me from being bored.” P1 “People aren’t waiting in the waiting room and not doing anything.”P1
Time to think	“The iPad helped [give us more time] to think about our answers.” P2 “Teenage brains can find it difficult to focus on their health needs, but the YouthCHAT gets them already thinking about their health and what they want to talk about before they come into see me, which was really helpful.” S1
Identifying issues	“The iPad helped pick up issues that I didn’t think I needed help with.” P4 “It helped me cut down and reflect on the multiple things I need help with.” P2 “It was definitely better at picking up acute issues specifically things around anxiety, drug and alcohol.” S2 “The results from the questionnaire would highlight things that may have not appeared in our standard consultations. It can identify what’s happening in the patient’s life in that point in time.” S1
Starting conversations/building rapport	“The doctor started the conversation after looking at the report.” P1 “It’s not awkward talking to them (the doctor and nurse).” P5 “It feels anonymous to them. They (patients) are not having to admit something to a doctor that they might find intimidating, and once we are made aware of whatever issue they have highlighted, it’s an opening for use to try and address that with them.” S1
Consultation Efficiency	“The standard holistic HEADSSS [Home, Education/Employment, Eating, Activities, Drugs and Alcohol, Sexuality, Suicide and Depression, Safety] assessment we are encouraged to use can take a long time to complete a good one. So use of the assessment tool can really look into managing our time well and also looking at other acute needs.” S2
Making Plans, Changes, Referrals	“It helped in entering into negotiating a plan. It sets the clinician in that mind frame that if a risk behaviour is present then what are the services that are available for this young person and can actually open up a conversation straight away.” S2
Patient-Clinician Relationship	“Some initial hesitations were whether the electronic tool removed the face to face engagement and connecting with young people. However, the outcome is that it hasn’t been hindered at all. I think it has enhanced it because we still do that engaging and connecting with young people and the YouthCHAT is an option for young people to feel more comfortable to answer questions.” S2

### Development of the implementation framework

**Fig. 3:**
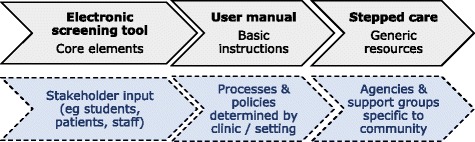
Implementation framework with core and modifiable components

There are core components necessary for the development of the electronic screening tool, its implementation in a practice or community setting, and the stepped-care resources provided for possible intervention for each domain. To enable the programme to be adaptable to real-life settings, and to be scaled up for utilisation in diverse contexts, the framework also needs to accommodate stakeholder regular input for each element of the process (see Fig. 3).

YouthCHAT was adapted from the generic eCHAT through input from, and piloting by, adolescents over a period of approximately nine months. Modifications requested by clinic staff, including sexual health questions, ability to select specific domains for screening and a Māori language option, were programmed into the tool. The WHO ASSIST was replaced by the youth-specific SACS for alcohol and drug screening. Along with in-person assistance, the YouthCHAT programme was implemented through development and utilisation of a comprehensive manual. User feedback helped clarify the instructions. Customisation of the programme (such as who is screened, frequency, which modules are used, mode of delivery including smart phone, electronic tablet or computer at clinic, school or home) is determined by each clinic or community setting, allowing tailoring for context.

Generic resources are provided for each domain, using a stepped care approach. For self-management these include educational brochures and other written resources, national helplines, web addresses and links to e-therapies. Clinicians are provided with details of possible medications and assistance with brief interventions. Clinics can enter their local community-based organisations and support agencies, and the relevant mental health and alcohol and drug secondary care services can be entered for their locality.

The current framework consists of a flexible tool, a comprehensive manual and resources with the ability to adapt to specific contexts to enable implementation to be scaled up.

## Discussion

YouthCHAT data in this study reveal participants to be an extremely vulnerable group, with 43 % smoking, 23 % with alcohol or drug issues, and 10 % with problem gambling. Comparable ‘CHAT’ studies with general practice patients aged 16 to 25 years found 29 % with smoking, 20 % with alcohol, 6 % with drug and 3 % with gambling issues, and considerably higher rates (33, 31, 30 and 10 % respectively) in international Asian students in New Zealand [16]. However these Māori youth display much greater desire for help than these previously studied groups.

YouthCHAT was demonstrated to be a useful and acceptable method for screening patients at a low decile school co-located clinic. Using YouthCHAT on the e-tablet to case-find and screen health problems was effective in allowing the young patients to think about their issues and talk about the results with their clinician. This was enhanced by the help question, which allowed patients to request intervention without the potential awkwardness of initial face-to-face dialogue. This aligns with other research that finds that consultations become more patient-centred when patients are empowered, with time to think and decide on whether to request help, thereby guiding clinicians and indicating their level of ‘readiness’ to participate in decision-making [17, 18]. Evidence indicates that this facilitates improved prioritisation and problem-solving, hence increasing consultation efficiency as well as enhanced patient health and self-management skills [19, 20].

Patients felt safe to answer questions on the e-tablet. Research shows that youth tend to be more involved and participatory when the medium for communication involves technology [21]. Technology also becomes a means of self-expression and engagement, hence increasing the comfort to be honest and provide more accurate health screening responses [22].

Early concerns have been raised that technology impairs rapport between patient and clinician by interference to visual, aural and/or tactile communication [23–25], and that technology has caused health care to be more profession than patient-centred [26]. However, this study indicates that YouthCHAT contributed to improved clinician-patient relationships. Only 6.5 % of patients were embarrassed to talk about their results with the clinician, and none felt judged for their responses, in line with previous work with this tool [10].

### Strengths and limitations

This study enabled the successful implementation of a youth screening and intervention program into a high-needs youth clinic, with adaption to local need. Comprehensive, systematic data of mental health and lifestyle issues and readiness to change facilitated patient/physician dialogue about appropriate intervention, and acceptability and utility were evaluated.

Limitations are the small sample size and specific clinic population with respect to possible generalisability. Attending patients were overwhelmingly female, hence this programme failed to cater for the large unmet need of young Māori males. While the clinic is available to both male and female youth, young Māori men in the community seldom present to primary care services, even when provided free. Young women tend to present for gynaecological reasons. More males do attend during the school year, when encouraged by school teaching and clinical staff. This highlights the need for out-reach services at places where young men congregate, including sports clubs, bars and marae (Māori meeting places). The study addressed feasibility and implementation, hence there was no control group and clinical outcomes data are not included. Funding is being sought for a clustered randomised trial to assess the clinical efficacy of YouthCHAT.

### Implications

Despite the high proportion of positive responses and requests for help, clinic staff valued YouthCHAT in facilitating good rapport and fostering the relationship by allowing joint decision-making and less interventionist care. Since the trial end-date, clinic staff continue to use YouthCHAT and have recommended it to other youth centres in the region. Planning is now underway for further implementation and upscaling into other youth clinics, tailoring the generic framework to specific contexts.

## Conclusions

This study used a community-based participatory research approach to engage key stakeholders (patients and clinic staff) for ‘real life’ translation of an electronic mental health and lifestyle screening and intervention package into a specific youth clinic context. Patients and staff found the programme acceptable and useful, and facilitated joint decision-making on possible interventions. A framework was developed for scaled up and sustainable implementation in other settings.

The researchers receive regular enquiries for the use and adaptation of the eCHAT programme in a variety of clinical and community settings, both within New Zealand and internationally. Research projects have been undertaken in Canada [27] and Hong Kong [28], and is under planning in Australia. Copyright for the programme sits with the researchers at the University of Auckland. Work in being undertaken to develop a licence for its use in different settings.

## Abbreviations

ASSIST: Alcohol, smoking and substance involvement screening test
CHAT: Case-finding and Help Assessment Tool
eCHAT: Electronic case-finding and help assessment tool
EMR: Electronic medical record
GAD-7: Generalised anxiety disorder
PHQ-9: Patient health questionnaire for depression
SACS: Substance and choices scale
